# Identification of protein coding regions in RNA transcripts

**DOI:** 10.1093/nar/gkv227

**Published:** 2015-04-13

**Authors:** Shiyuyun Tang, Alexandre Lomsadze, Mark Borodovsky

**Affiliations:** 1 School of Biology, Georgia Institute of Technology, Atlanta, GA 30332, USA; 2 Joint Georgia Tech and Emory Wallace H. Coulter Department of Biomedical Engineering, Georgia Institute of Technology, Atlanta, GA 30332, USA; 3 School of Computational Science and Engineering, Georgia Institute of Technology, Atlanta, GA 30332, USA; 4 Center for Bioinformatics and Computational Genomics, Georgia Institute of Technology, Atlanta, GA 30332, USA; 5 Department of Biological and Medical Physics, Moscow Institute of Physics and Technology, Moscow, Russia

## Abstract

Massive parallel sequencing of RNA transcripts by next-generation technology (RNA-Seq) generates critically important data for eukaryotic gene discovery. Gene finding in transcripts can be done by statistical (alignment-free) as well as by alignment-based methods. We describe a new tool, GeneMarkS-T, for *ab initio* identification of protein-coding regions in RNA transcripts. The algorithm parameters are estimated by unsupervised training which makes unnecessary manually curated preparation of training sets. We demonstrate that (i) the unsupervised training is robust with respect to the presence of transcripts assembly errors and (ii) the accuracy of GeneMarkS-T in identifying protein-coding regions and, particularly, in predicting translation initiation sites in modelled as well as in assembled transcripts compares favourably to other existing methods.

## INTRODUCTION

Prior to the advent of next-generation sequencing (NGS), transcriptome data were scarce and limited to full messenger RNA (mRNA) and expressed sequence tag (EST) libraries covering at best a few hundred genes of a given species (1). RNA-Seq technology (2) generates a vast number of short reads that must be assembled into transcripts. Several computational methods were developed for transcript reconstruction by short read assembly (assessed in (3)). The important next step in transcript downstream analysis is transcript annotation, particularly identification of protein-coding regions.

Finding genes in transcripts by mapping known proteins can be successfully implemented only if the protein products of encoded genes have homologs in protein databases. Discovery of novel genes requires methods that are alignment-free. Earlier developed *ab initio* gene prediction methods for EST and complementary DNA (cDNA) sequences, such as ESTscan (4), used hidden Markov models (HMM) and required curated training sequences for estimation of model parameters. The supervised training protocol adds downtime that makes application of such tools less practical. The support vector machine based method names “CONC" (5) was developed to identify transcripts that contain protein-coding genes and discriminate them from non-translatable transcripts. Since CONC does not parse transcripts into coding and non-coding regions we were not able to use this method in comparisons of gene prediction tools where we have to compare predicted gene borders. A recent *ab initio* tool, TransDecoder, a companion of the *de novo* transcriptome assembler Trinity (6), generates the training set by a simple automatic procedure that identifies long open reading frames (ORFs) in the assembled transcripts.

Self-training has already been used in algorithms for *ab initio* gene finding in prokaryotic genomes, particularly in the frequently used GeneMarkS (7), Prodigal (8,9) and Glimmer3 (8,9). Since one of the Prodigal modes (‘switched-off RBS model’) can be used to predict intronless genes in eukaryotic transcripts we included this method in the modelling and analysis of gene prediction in transcripts.

Presented here GeneMarkS-T extends prokaryotic GeneMarkS (7) to prediction of continuous (intronless) protein-coding regions in eukaryotic transcripts. We assume that a correctly spliced and reconstructed eukaryotic transcript should carry a single functional protein-coding gene. Two or more genes in a single transcript would make an operon structure typical for bacteria. With few exceptions eukaryotes possess no operon organization. When several protein-coding genes are predicted in a single transcript one could think about either biological (e.g. presence of alternative isoforms) or technological reasons (e.g. erroneous assembly). When two or more protein-coding regions are predicted, GeneMarkS-T assigns a log-odds score to each prediction. We show that the gene with the max log-odds score in a given correctly assembled transcript has a high likelihood to be the true gene.

Transcriptomes of large eukaryotic genomes may exhibit significant variation in nucleotide composition. This inhomogeneity complicates algorithm training and affects the accuracy of gene prediction. GeneMarkS-T divides the whole set of transcripts into several sets (clusters) more homogeneous in G + C composition and derives several cluster-specific models of protein-coding regions.

Accurate identification of the translation initiation site (TIS) is not a simple task. Although it is often assumed that the 5′-most AUG codon in a protein coding ORF serves as the true TIS, this is not always the case. True TISs were shown to appear in the sequence context known as the Kozak pattern (10) with relatively weak positional preference for certain nucleotides around the AUG codon. The assessment of accuracy of TIS predictions requires a sufficient number of genes with experimentally verified TIS positions. The recently introduced ribosome profiling, the Ribo-seq technique (11), makes it possible to use deep sequencing of mRNA fragments protected by initiating ribosomes (12) to generate large sets of genes with verified TIS positions. We use mouse transcripts with TIS annotation derived from the Ribo-seq experiments as a test set to determine accuracy of TIS predictions.

Other test sets that we used in our computational experiments for comparative assessment of gene prediction tools were (i) the sets of reference transcripts of the fission yeast, *Schizosaccharomyces pombe*the mustard plant, *Arabidopsis thaliana*, the fruit fly, *Drosophila melanogaster* and the rodent, *Mus musculus*; (ii) sets of *D. melanogaster* transcripts assembled from RNA-Seq reads by five tools that participated in the RGASP competition (3). Along the way we have shown that assembly errors in real transcripts do not have any noticeable effect on the GeneMarkS-T unsupervised training.

When we studied the accuracy of GeneMarkS-T with respect to variation in volume of training sequence we have shown that even for the small size training sets, approaching the size of a single transcript, the accuracy of the tool remains high. This feature makes GeneMarkS-T a suitable tool for gene prediction in metatranscriptomes.

## MATERIALS AND METHODS

### Algorithm design

The GeneMarkS-T and GeneMarkS (7) algorithms share the following: (i) the *heuristic* method of initialization of the hidden semi-Markov model (HSMM) parameters (13), (ii) the Viterbi algorithm that finds maximum likelihood parse of transcript sequence into coding and non-coding regions and (iii) the concept of iterative self-training (7).

**Figure 1.**
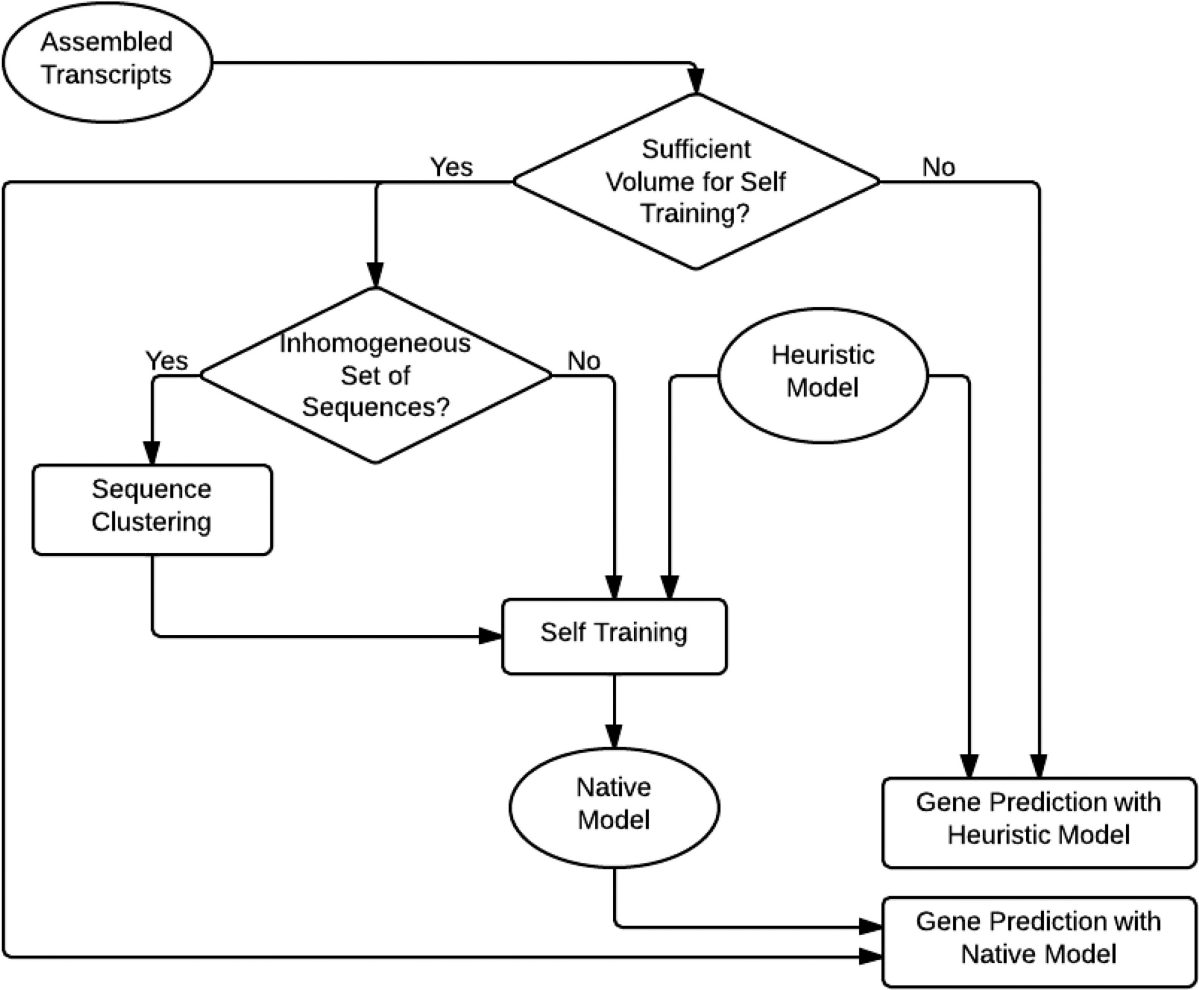
Flowchart of the GeneMarkS-T model training and gene prediction steps.

Important differences are as follows. Unlike rather homogeneous G + C content of prokaryotic genomes, variation in local G + C content across much longer eukaryotic genomes may reach 30–40%. It was shown that genomic sequence G+C content is one of the major factors driving the genome-wide pattern of codon usage (13,14). Therefore, GeneMarkS-T attempts to group transcripts by G + C content (Figure 1). The number of groups (clusters) depends on how wide is the distribution of G + C composition of the whole set of transcripts. By adjusting cluster borders we place the same volume of transcript sequence into each cluster. The iterative self-training on sequences of each cluster runs similarly to that described for GeneMarkS (7). The procedure starts with initialization of the cluster-specific ‘heuristic’ model (13). Then rounds of (i) predictions of protein-coding regions, (ii) selecting a new set of sequences of predicted genes for training and iii/ re-estimation of parameters, follow until convergence, i.e. the set of predicted genes in the last iteration should be the same as in the previous iteration (Figure 1).

The total volume of transcript data may vary. If the input data is not large enough for self-training, the ‘heuristic’ parameters used in initialization (13) are accepted as the final set of parameters and predictions made with this parameter set are considered final. The rationale for this approach is the earlier demonstration that the ‘heuristic’ parameters give sufficiently accurate predictions of continuous protein-coding regions in short prokaryotic sequences, e.g. in metagenomic sequences (13,15).

GeneMarkS-T derives in iterations the species-specific Kozak pattern, positional frequency model of the sequence near TIS (10). The frequencies are determined from the multiple alignment of 12 bp-long fragments surrounding predicted TISs with nucleotides A of start codons situated in position 7.

Recently introduced strand-specific RNA-Seq technology (16) determines which DNA strand served as a template for transcription. If this information is available GeneMarkS-T changes the HSMM architecture and eliminates states related to the non-transcribed DNA strand; this change reduces the rate of false positive predictions. In what follows GeneMarkS-T version with the strand specific HSMM is designated as GeneMarkS-T(S).

For genes predicted in each transcript GeneMarkS-T assigns log-odds scores computed as log of the ratio of probability of a sequence given the coding model to probability of the same sequence given the non-coding model. The distribution of lengths of protein coding region and non-coding sequences is taken into account; these distributions are modelled as the gamma distribution the exponential distribution, respectively (17).

### Test set preparation

We used annotated mRNA sequences of *A. thaliana*. *D. melanogaster, M. musculus* and *S. pombe* as a set of ‘complete’ reference transcripts. We screened the RefSeq mRNA sequences with prefixes ‘NM_’ indicating curated records. Records with no annotation for a start or stop codon, with annotated frameshifts, or with stop codon read-through were eliminated. We also removed records with no annotated UTRs, which were likely to be generated by computational prediction. From mouse and fly transcripts representing alternative isoforms of the same gene only one isoform, selected at random, was kept per gene. The numbers of initial RefSeq sequences and the numbers of sequences in the final sets of ‘complete’ reference transcripts are given in Table 1.

**Table 1.** Composition of the test sets of ‘complete’ reference transcripts

Species	No. of mRNAs in RefSeq database^a^	No. of curated records with ‘NM_’ prefix	No. of transcripts after filtering (see ‘Mateials and Methods’ section)
*S. pombe*	5123	4841	4655
*M. musculus*	77 925	28 887	18 937
*D. melanogaster*	30 264	30 264	13 241
*A. thaliana*	35 173	35 173	28 805

^a^October 2014.

Preparation of sets of *assembled* transcripts was facilitated by the published study of the RGASP competition; the latest study of the accuracy of transcript reconstruction from RNA-Seq reads (3). Transcripts could be assembled by different tools; we used five sets of *D. melanogaster* transcripts generated in (3) by Cufflinks (18), Augustus (19), Velvet (20), Oases (21) and Exonerate (22) from co-ordinates of the exons of assembled transcripts as provided by the authors of (3). Additionally we constructed a set of 24 804 *reference* transcripts of *D. melanogaster* with respect to the FlyBase genome annotation (FB2013_01); the same set was used in RGASP competition for comparison with the *assembled* transcripts (3). We removed 350 transcripts containing incomplete genes or some non-canonical features (frameshifts or stop codon read-through), 70 pseudo genes, and 786 non-protein-coding RNAs (ncRNA, tRNA, snoRNA, etc). The final set with 23 598 reference transcripts was used for identification of the features of sequence assembly and for assessment of the accuracy of gene prediction (Supplementary Figures S1–S3 and Table 2).

**Table 2.** Results of assessment of gene prediction accuracy of GeneMarkS-T, Prodigal and TransDecoder on 1392 mouse transcripts with experimentally verified translation initiation sites (annotated CDS length >300 bp; *mgl* for all tools 300 bp)

	Exact 3′ end	Exact 5′ and 3′ ends	#FP	#Shorter	#Longer
Prodigal	1193 (85.7%)	612 (51.3%)	351	9	572 (571)
TransDecoder	1193 (85.7%)	623 (52.2%)	428	0	570 (568)
GeneMarkS-T	**1197** (**86.0%**)	**821** (**68.6%**)	**195**	43	333 (333)
GeneMarkS-T^1^	1196 (85.9%)	694 (58.0%)	196	51	451 (450)
GeneMarkS-T^2^	1194 (85.8%)	1134 (95.0%)	197	59	1 (0)
GeneMarkS-T^3^	1147 (82.4%)	630 (54.9%)	321	259	258 (204)

The columns show (from left to right) the number of genes (i) with 3′ ends correctly identified and its fraction (%) in the whole set of transcripts; (ii) exactly predicted (both 5′ and 3′ ends correctly identified) and its fraction (%) among genes with correctly predicted 3′ ends; (iii) not matching annotation in 3′ end (false positives); (iv) predicted shorter than annotated; (v) predicted longer than annotated, with number of predicted genes with 5′ end beyond the 5′ border of actual transcript sequence (incomplete predictions) shown in parentheses. The results are also shown for GeneMarkS-T runs ^1^ without model for the Kozak motif; ^2^ with requirement to predict 5′ complete genes; ^3^ analyzing each transcript independently with use of only one iteration; parameters of heuristic models for each transcript were selected as functions of the given transcript G+C content (simulation of a run on metatranscriptome with 1147 sequences).

We also used comparative analysis of the assembled transcripts and the reference transcripts for coming up with a realistic dataset of ‘partial’ reference transcripts. First, we aligned the *D. melanogaster* transcripts assembled by the five tools to the corresponding *D. melanogaster* reference transcripts (3). Next, we determined how frequently a given part of reference transcript was present in the assembled transcript (Supplementary Figure S1B). This analysis demonstrated that it is common to observe partial transcripts depleted on both ends. Therefore, we simulated partial transcripts of four species by trimming complete reference transcripts at each end with frequency determined by the experimentally observed distribution (Supplementary Figure S1B).

To assess accuracy of TIS prediction, we used a set of complete transcripts of *M. musculus* with TIS annotated in Ribo-seq experiments (12). We used a conservative approach and first selected 1455 transcripts where the Ribo-seq data confirmed TIS matched the annotated TIS; further on we removed transcripts with annotated genes shorter than 300 bp and ended up with 1392 transcripts used in the tests.

### Aligning assembled and reference transcripts

The *D. melanogaster* transcripts reconstructed by the five tools were aligned to the reference transcripts by BLASTn. Since both the assembled transcripts and the reference transcripts were defined via exon co-ordinates on genomic sequence (FB2013_01 in FlyBase) we required 100% nucleotide identity in the alignments.

An assembled transcript was classified as ‘concordant’ if it had a section that could be aligned without gaps to the whole coding region (or to its continuous part) in the reference transcript (Supplementary Figure S2a–c). The alignment identity was not traced in the UTR sections of reference transcripts. Still the length of the ‘UTR section’ of an assembled transcript (situated upstream or downstream of the ‘coding’ section aligned to the reference transcript coding region) was required not to exceed the length of reference UTR by more than 300 bp (Supplementary Figure S2c). An assembled transcript was classified as ‘conflicting’ if it did not have a section that could be aligned without gaps to the CDS of reference transcripts (Supplementary Figure S2d–f), or the UTRs of assembled transcripts were longer than the reference UTR(s) by 300 bp (Supplementary Figure S2g). Assembled transcripts that could not be aligned to references with *E*-values better than 0.001 were classified as ‘not-aligned’.

### Assessment of gene prediction accuracy

Along with GeneMarkS-T we assessed the performance of specialized tools for gene prediction in transcripts: ESTscan, v2.1 (4), TransDecoder (http://transdecoder.sourceforge.net), as well as prokaryotic Prodigal, v2.60 (8) used in the special mode of predicting ‘intronless genes’.

The accuracy of gene prediction in the test sets was determined by comparison with annotation. A prediction that correctly identified the reading frame was treated as a true positive prediction (TP); a correctly predicted reading frame would entail an exact match of predicted and annotated stop codons (for 3′ end complete genes). Sensitivity (Sn) and specificity (Sp) of a whole set of predictions was computed as Sn = #TP/(#TP + #FN) and Sp = #TP/(#TP + #FP), respectively, where #FN stands for the number of false negative and #FP stands for the number of false positive predictions.

We classify a predicted coding region as ‘false positive’ if it does not match the annotation (in the sense of match of 3′ ends). Here, we have to say that, in general, computational science operates with sets of true and false objects to evaluate classification algorithms. This approach is difficult to implement in full in genome analysis and, particularly, in gene prediction. We do use the true set, the set of annotated genes. However, we do not have a verified set of ‘non-genes’. It is difficult to prove experimentally that a particular segment of a nucleotide sequence is not expressed as a part of a protein coding gene. Therefore, what we use essentially as surrogate ‘non-genes’ are the sequences of open reading frames that are not annotated as genes.

In the test runs, all the parameters of each gene finding tool were set to default values except for the *mgl* (minimal gene length) threshold. The *mgl* threshold changes the balance between Sn and Sp; the *mgl* value was varied to generate ROC-like dependencies (see ‘Results’ section). If the *mgl* value was not among adjustable settings, as in Prodigal, predicted genes shorter than the selected *mgl* were filtered out in post-processing.

GeneMarkS-T and TransDecoder have standard ‘strand specific’ options for analysing transcripts generated by assembly of stranded RNA-Seq reads. To emulate such an option for Prodigal we filtered out protein-coding regions predicted in the designated complementary strand.

## RESULTS

### Accuracy of gene prediction in reference transcripts

**Figure 2.**
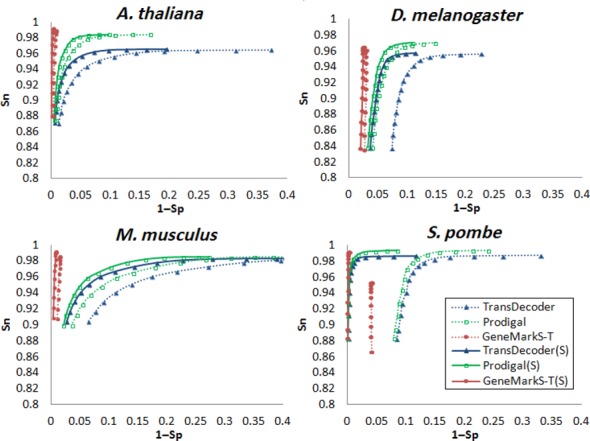
Plots of gene prediction sensitivity (Sn) as functions of gene prediction specificity (1 − Sp) for TransDecoder, Prodigal and GeneMarkS-T determined on test sets of ‘complete’ reference transcripts of *A. thaliana, D. melanogaster, M. musculus* and *S. pombe*. We applied the three tools in both strand blind and strand informed (S) modes. To build the curves we generated sets of predicted genes with minimal length controlled by the *mgl* threshold (see text). As the *mgl* values increased from 90 to 480 bp (with 30 bp step) the Sn values decreased.

**Figure 3.**
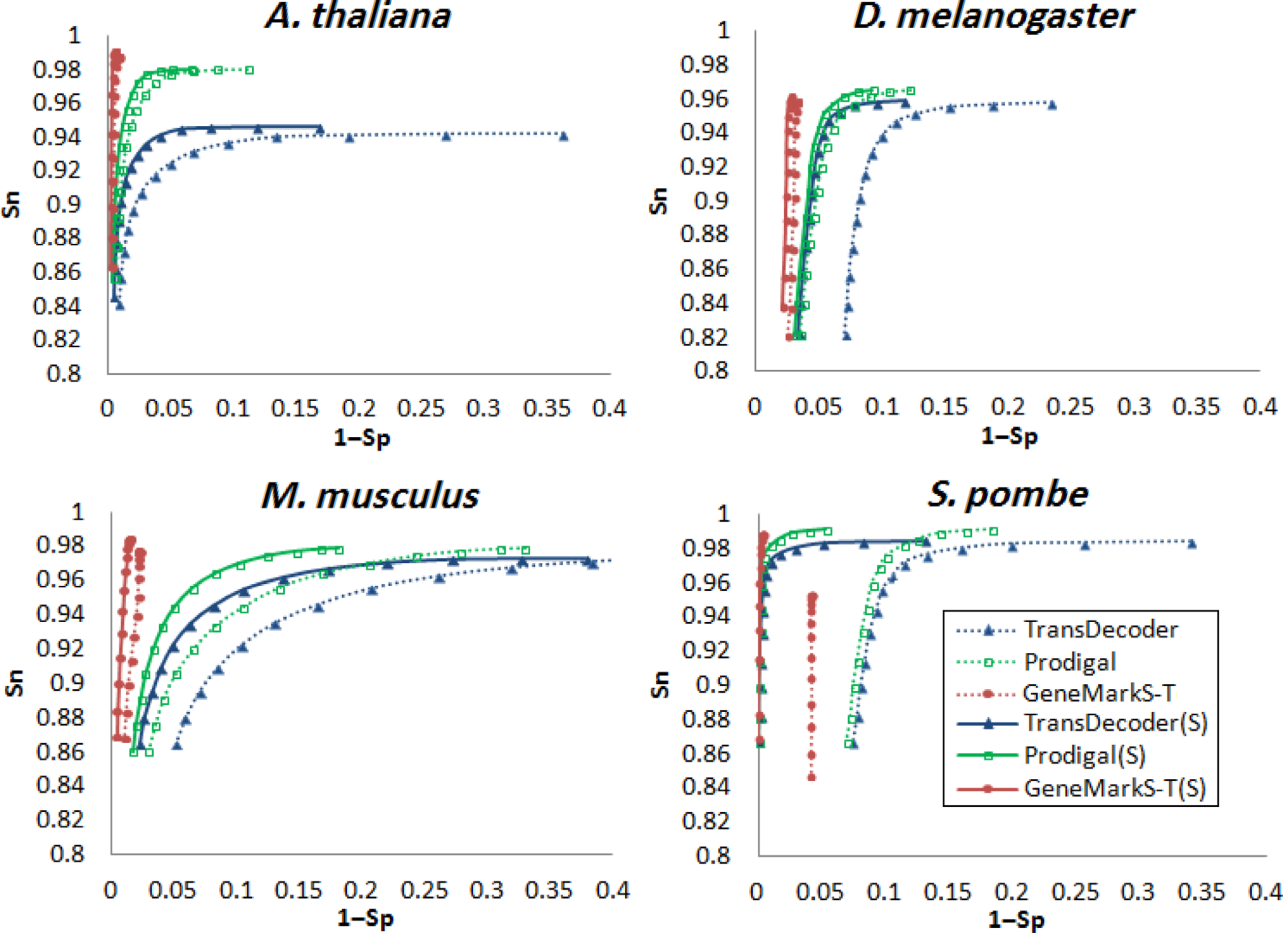
Same as in Figure 2 for the tests on simulated ‘partial’ reference transcripts of *A. thaliana, D. melanogaster, M. musculus* and *S. pombe*. The ‘partial’ transcripts were made by trimming sequences on both 5′ and 3′ end of the ‘complete’ transcripts (see text for rational of this method). The three tools were used in both strand blind and strand informed (S) modes.

We used GeneMarkS-T, Prodigal, TransDecoder and ESTscan to predict protein-coding genes in ‘complete’ as well as ‘partial’ transcripts of *A. thaliana, D. melanogaster, M. musculus* and *S. pombe* (see ‘Materials and Methods’ section). The number of genes predicted in a set of transcripts depends on the selected minimum gene length (*mgl*). We have changed *mgl* as a threshold parameter from 90 to 480 bp (with 30 bp steps). For each set of predictions we computed Sn and Sp based on the transcript annotation and plotted the dependence of Sn on 1 − Sp (Figures 2 and 3). In these plots, which look similar to receiver operating characteristic (ROC) curves, the top right points were obtained for *mgl* equal to 90 bp. We do not show plots for ESTscan as we were not able to achieve high enough performance (i.e. for mouse we had Sn = 0.53 and Sp = 0.54). We believe that self-training would improve ESTscan performance. In the absence of such an option we were forced to select one of the available pre-defined models, e.g. the human model for analysis of mouse transcripts.

For ‘complete’ transcripts, both strand-blind and strand-specific versions of GeneMarkS-T demonstrated significantly better performance than the other tools (Figure 2). In experiments with ‘partial’ transcripts (Figure 3) Prodigal and TransDecoder came closer in performance to GeneMarkS-T. The best (Sn + Sp)/2 we saw for GeneMarkS-T, Prodigal and TransDecoder when the *mgl* values were 150, 210 and 270 bp, respectively. Adding information on RNA strand and thus use of the (S) versions of the three gene finding tools, increased the Sp values (Figures 2 and 3).

Significant variation in G + C content in *M. musculus* and *D. melanogaster* transcripts (from 0.31 to 0.76 in mouse and from 0.27 to 0.63 in fly) was immediately identified by GeneMarkS-T which grouped the transcripts into three G + C content bins with automatically defined borders (Table S1). Self-training was done separately for transcripts in each of the three clusters. In the prediction step, algorithm parameters used for a given transcript were chosen with respect to the transcript G + C content. This approach produced better Sn values than in the absence of clustering (Table S1).

**Figure 4.**
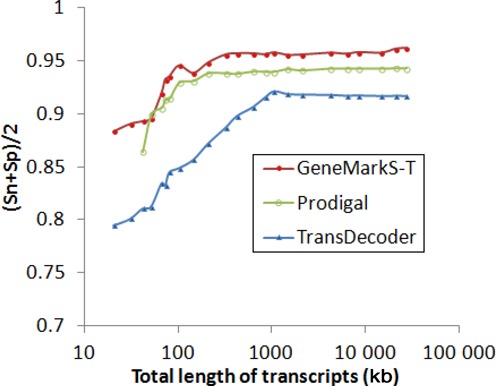
Dependence of (Sn + Sp)/2 of the three gene prediction tools on the size of training set of *D. melanogaster* transcripts (X axis shows the total set size, log scale). Sets of transcripts of the same size were sampled randomly 50 times from the whole set of reference transcripts. The *mgl* value that achieved best overall (Sn + Sp)/2 was tool specific (150 bp for GeneMarkS-T, 210 bp for Prodigal and 270 bp for TransDecoder).

We studied how prediction accuracy depends on the volume of transcripts in training. For these experiments we sampled randomly several sets of transcripts with the same volume. If the volume was larger than 600 kb, GeneMarkS-T and Prodigal reached a plateau with steady performance and (Sn + Sp)/2 value close to 96% for GeneMarkS-T and 94% for Prodigal (Figure 4). Accuracy of TransDecoder had a similar pattern of change with the plateau at 91% reached at the volume of 1 Mb. A decrease to 100 kb produced lower but still decent performance: 90% for GeneMarkS-T and Prodigal, and 80% for TransDecoder. The minimum volume of sequence required for Prodigal was 20 kb while the GeneMarkS-T limit was even lower. Below 50 kb GeneMarkS-T automatically switches to use of heuristic models of protein-coding regions whose parameters could be determined for a sequence fragment as short as 400 bp (15).

**Figure 5.**
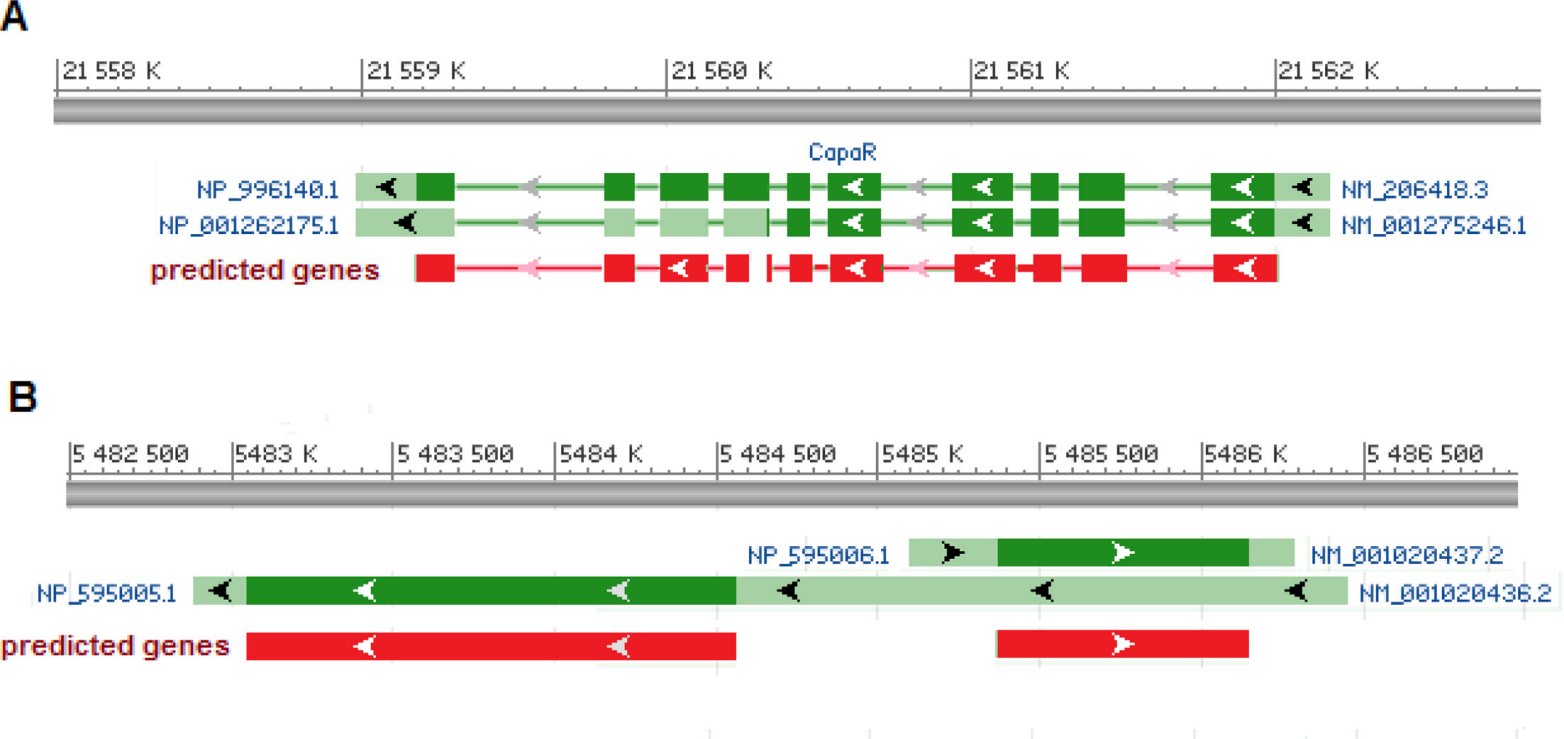
Diagrams of two typical events when more than one coding region is predicted in a transcript. We show pre-spliced transcripts: genomic sequences are shown as grey bars; exons defined by annotation are shown as wider bars (green colour—UTR, dark green—CDS); predicted protein-coding exons are shown as red bars. (**A**) Two transcripts are originated from the same location of *D. melanogaster* genome (NM_001275246.1 and NM_206418.3). The FP prediction (the downstream gene in complementary strand) is a part of the coding region of alternative isoform of CapaR gene. (**B**) The 5′ UTR of *S. pombe* transcript NM_001020436.2 overlaps with another transcript NM_001020437.2 originated from complementary strand. This transcript topology leads to two predictions in transcript NM_001020436.2: one in the direct strand (FP) as well as one in the complementary strand (TP). The figures were made with the NCBI RefSeq sequence viewer.

In some transcripts GeneMarkS-T predicted several coding regions (with *mgl* 300 bp). We observed such outcomes in 2.5% of *A. thaliana* transcripts, 9.4% of *S. pombe*, 6.0% of *D. melanogaster* and 20.4% of *M. musculus*. In the supposed absence of operons such outcomes are possible for three reasons. First, additional predictions could have no connection to carrying genetic code, i.e. pure false positives. Second, a transcript could come from a locus where splicing mechanism generates alternative isoforms. For instance, protein-coding exons related to one isoform could appear outside the protein coding region related to another isoform (e.g. Figure 5A). Third, a transcript could overlap adjacent genes located in the complementary strand. Particularly, *S. pombe*, a species not known for ubiquitous alternative splicing, has short intergenic regions and long UTRs that may overlap adjacent genes (e.g. Figure 5B). Not surprisingly, for *S. pombe* we observed a significant gain of accuracy after switching to strand-specific versions of the three gene finders (Figures 2 and 3).

If multiple predictions were generated in a transcript GeneMarkS-T selected the one with the maximum log-odd score. This approach produced 93% success rate in selecting the ‘true’ coding region for *A. thaliana*, 74% for *D. melanogaster*, 98% for *M. musculus* and 62% for *S. pombe*.

### Prediction of translation initiation site

To assess the accuracy of TIS prediction by GeneMarkS-T, Prodigal and TransDecoder we used 1392 reference transcripts of *M. musculus* (with annotated coding regions longer than 300 bp). The TIS annotation in these transcripts was validated by Ribo-seq experiments (see ‘Materials and Methods’ section). GeneMarkS-T was run in three modes: (i) with default settings; (ii) with search for the Kozak motif switched off; and iii/ with mandatory prediction of complete CDS.

GeneMarkS-T with default settings correctly predicted 68.5% starts in genes where the reading frame was correctly predicted (and, therefore, the 3′ end of the gene). This was higher accuracy in comparison with the two other tools (Table 2). All three tools revealed a tendency to extend the 5′ end of the coding region beyond the 5′ end of the transcript. Notably, TransDecoder adopts the ‘longest-ORF’ rule and selects the 5′-most AUG (with respect to the in-frame stop codon) as the translation initiation site. In comparison, GeneMarkS-T had the largest fraction of TIS predictions located downstream from the 5′-most AUGs. Use of the Kozak motif was responsible for improving Sn of GeneMarkS-T by about 10% (Table 2). Prohibiting predictions of incomplete coding regions would boost the TIS identification accuracy of GeneMarkS-T to 95.0%, however, use of this option is limited to transcripts that are known to be 5′ end complete.

**Table 3.** Numbers of protein-coding regions predicted correctly (TP) and incorrectly (FP) by GeneMarkS-T, Prodigal and TransDecoder in *D. melanogaster* ‘concordant’ transcripts (selected as described in text)

Transcripts built by	No. of transcripts	GeneMarkS-T		Prodigal		TransDecoder	
		TP	FP	TP	FP	TP	FP
Cufflinks	7222	**7162**	**60**	7098	232	7046	432
Augustus	9444	**9423**	**21**	9383	246	9332	480
Exonerate	6971	**6953**	**18**	6940	190	6915	454
Velvet	7344	**7146**	**198**	7096	312	7030	429
Oases	13 869	**13 769**	**100**	13 659	347	13 598	582

Predictions shorter than the tool-specific *mgl* (150 bp for GeneMarkS-T, 210 bp for Prodigal and 270 bp for TransDecoder) were filtered out. Bold font highlights best results in a particular row (the largest TP and the smallest FP).

Several ribosome profiling studies (12,23–24) raised concerns about the frequent presence of alternative TIS's located both upstream and downstream of annotated TIS's confirmed by Ribo-seq experiments. However, a recent publication (25) indicated that reports of alternative TIS in many cases are likely to be artefacts; therefore, the confidence in the Ribo-seq experimental validation of annotated TIS's remains high.

### Gene prediction with heuristic models (case for meta-transcriptomics)

To model gene prediction in a metatranscriptome we used the same set of mouse transcripts; G + C content of individual transcripts in this set ranged from 27 to 63%. To run GeneMarkS-T on a given transcript we used parameters derived as functions of a single variable, the transcript G + C content. We did not continue the training, assuming that the given transcript is the only sequence from an unknown genome. This assumption is relevant for a typical metatranscriptome. The method of inference of these functions was described earlier for short metagenomics sequences (7,15). We used the functions that reflect dependence of oligonucleotide composition of protein coding regions on G + C content of the sequence; the functions were derived for a set of complete prokaryotic genomes (15). The results are surprisingly good (last row in Table 2); with correct prediction of 82.4% of genes (1147 out of 1193); also 54.9% of starts were correctly predicted in comparison with 68.6% correct starts predicted with full training of the model.

### Model training and gene predictions for transcripts reconstructed from RNA-Seq

**Figure 6.**
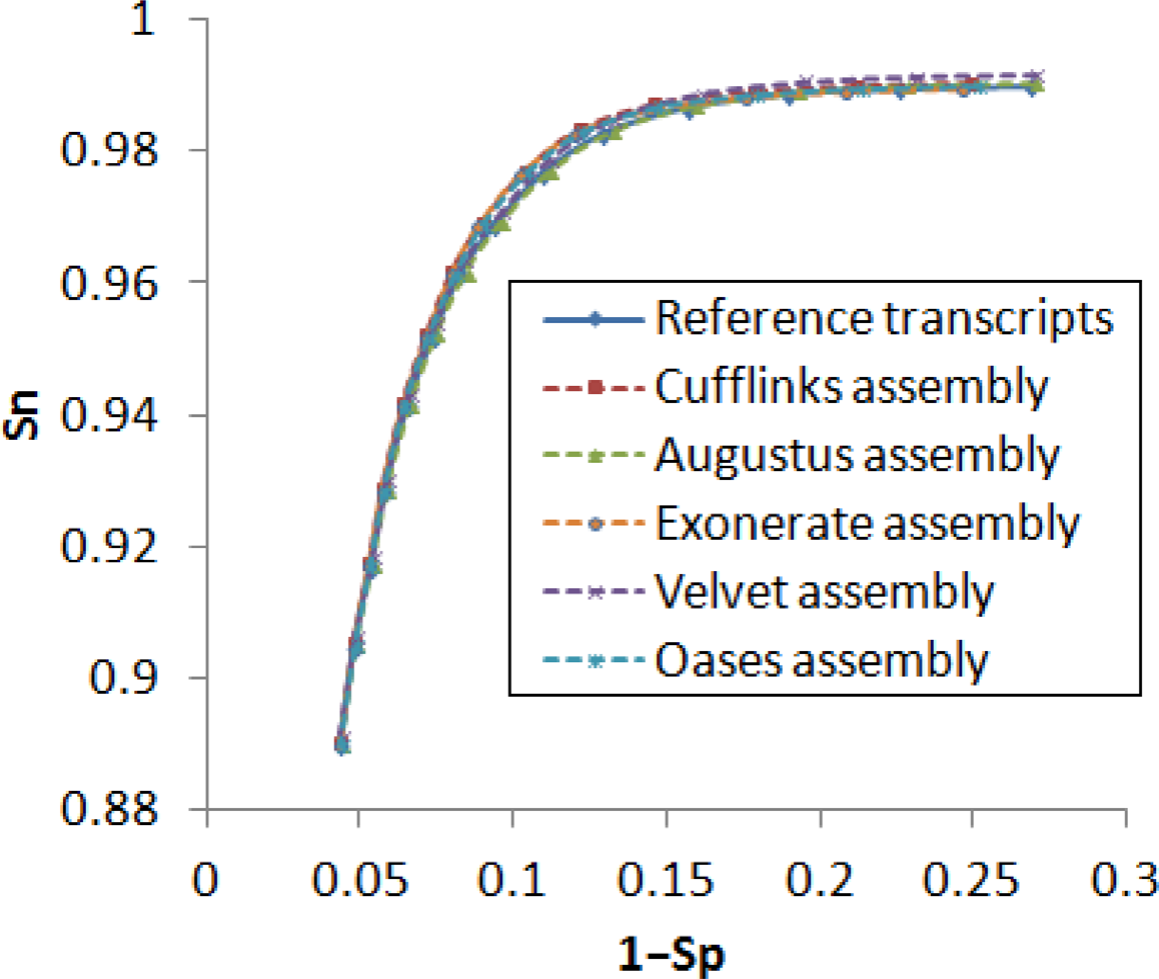
Plots of gene prediction accuracy in *D. melanogaster* reference transcripts built for GeneMarkS-T trained on sets of different types. The models were trained either on the set of *D. melanogaster* reference transcripts or on the sets of transcripts assembled by the five transcript assembly tools. Predictions made in reference transcripts were compared with annotation.

A comprehensive assessment of the accuracy of transcript reconstruction from RNA-Seq reads was conducted in the RGASP competition (3). We used in this study transcripts reconstructed in (3) by Cufflinks, Augustus, Exonerate, Velvet and Oases (18–22). It was shown that assembled transcripts frequently contain errors and only a subset of all transcripts could be fully recovered (3). Observed average lengths of assembled transcripts were shorter than that of reference transcripts, particularly the average lengths of the *de novo* assemblies made by Oases and Velvet (Supplementary Figure S1A). Would the errors present in transcript assemblies affect self-training of GeneMarkS-T? To address this question we trained GeneMarkS-T on five sets of *D. melanogaster* transcripts assembled by the five tools mentioned above. The trained models were used in GeneMarkS-T to predict genes in reference transcripts of *D. melanogaster*. We observed almost no difference between any of the five graphs of Sn versus 1 − Sp for gene prediction with models trained on *D. melanogaster* assembled transcripts and the graph depicting Sn versus 1 − Sp for gene prediction with the *D. melanogaster* model trained on reference transcripts (Figure 6). Thus, GeneMarkS-T training was shown to be robust with respect to use of assembled transcripts instead of ‘ideal’ reference transcripts.

To assess performance of gene prediction methods in assembled transcripts we used the same five sets of assembled *D. melanogaster* transcripts. First, we mapped the assembled transcripts to the corresponding reference transcripts (3) to detect and evaluate the differences. We used the results to divide the set of assembled transcripts into three groups: ‘concordant’, ‘conflicting’ and ‘not-aligned’ (see ‘Materials and Methods’ section and Supplementary Figure S2). Many assembled *D. melanogaster* transcripts fell into ‘conflicting’ category (from 17 to 47%, depending on the tool, see Supplementary Figure S3, ‘A’ bars); Cufflinks, Exonerate and Oases produced larger numbers of ‘conflicting’ transcripts than Augustus and Velvet. Multiple protein-coding regions were predicted more frequently in the ‘conflicting’ transcripts than in the ‘concordant’ transcripts (Supplementary Figure S4). Note, that for GeneMarkS-T events of prediction of multiple coding regions were registered prior to selecting ‘reported’ predictions with highest log-odd score. We have illustrated the distribution of events (multiple, single, none predictions) for GeneMarkS-T (Supplementary Figure S4). The distributions of the same events for the two other gene prediction tools show similar patterns (Table S2). Thus, all the tools predict single coding regions in ‘concordant’ assemblies with higher frequencies than in ‘conflicting’ ones.

To make unambiguous comparison of accuracy of gene prediction in ‘concordant’ transcripts we had to select the sets where gene finders make single gene predictions. As such surrogate sets we chose sets of ‘concordant’ assemblies where GeneMarkS-T predicted single protein-coding regions. Annotation of protein coding regions in these assembled transcripts was accomplished by transfer of the reference transcript annotation. In all the five test sets, GeneMarkS-T generated the largest number of TPs and the fewest number of FPs (Table 3).

In the sets of assembled transcripts where GeneMarkS-T predicted multiple coding regions we have observed high fractions of ‘conflicting’ transcripts (e.g. 90%, for the set of Cufflinks assembled transcripts). Thus, predicting multiple coding regions was an indicator of a higher chance for the transcript to be in the ‘conflicting’ category and to carry some discrepancies in the transcript assembly. Still, this observation should be taken with a caveat that multiple coding regions could appear in the ‘concordant’ transcript encoding alternative isoforms (as illustrated in Figure 5).

Very short coding regions are rare and are rarely predicted. Therefore, if an assembled transcript (complete or incomplete) is short it is likely that no gene will be predicted. Indeed, we observed that the gene finding tools did not predict genes in many transcripts assembled by the *de novo* methods Velvet and Oases (Supplementary Figure S3). Notably, many of these transcripts were too short (Supplementary Figure S1A).

## DISCUSSION

*Ab initio* gene prediction in transcripts has rarely been addressed compared to gene prediction on the genomic level. At the time of Sanger sequencing, annotation of Sanger transcripts (ESTs) was frequently done by alignment based methods as well as experimentally. With the advent of NGS and RNA-Seq the GenBank submission is concerned mainly about sets of reads, making it appear that transcript assembly and annotation is a background task. Still, accurate annotation of eukaryotic transcripts has become important in projects whose goal is restricted to generating transcript data. Also, traditional genome projects now frequently add a transcriptome sequencing component. Tools of gene prediction in transcripts provide, at least in theory, benefits of much more accurate identification of starts and stops of translation of a gene than tools predicting the same sites in a genomic context. When found in short exons, such sites are notoriously difficult to pinpoint in genomic sequence.

Despite the theoretical benefits, we have demonstrated that gene prediction in assembled transcripts faces challenges. We observed multiple gene predictions in single *assembled* transcripts to be much more frequent than in *reference* transcripts (Table S2). We demonstrated that presence of RNA-Seq assembly errors was the main cause of multiple predictions. The RGASP competition has shown that the species-specific error rates in transcript assembly are quite high (3). For instance, in the tests of assembly tools on the sets of RNA-Seq reads from the three transcriptomes, *Homo sapiens, Caenorhabditis elegans* and *D. melanogaster*, the highest percentage of correct transcript assembly (observed for *C. elegans*) was only 48% (3).

In our tests, we used the data from the RGASP project (3) and divided the whole set of transcripts into ‘concordant’, ‘conflicting’ or ‘not-aligned’ (see ‘Materials and Methods’ section). This division was useful in many respects. Particularly, we observed that multiple protein-coding regions were predicted in ‘conflicting’ transcripts much more frequently than in ‘concordant’ transcripts. Given that eukaryotes, as a rule, do not have operons, the multiple predictions should signal either the presence of parts of other genes (adjacent or alternatively spliced) or the presence of assembly errors. We observed that the latter outcome has high frequency, e.g. if multiple CDS are predicted in a Cufflinks assembled transcript then the chance for the transcript to belong to ‘conflicting’ or ‘not-aligned’ category is 93% (Supplementary Figure S4). Therefore, prediction of multiple genes in a transcript could serve as indicator of likely erroneous assembly.

Classification of the transcripts where multiple CDS were predicted is a special topic. For instance, tools such as GeneTack (26-28) could identify frameshifts that disrupted a single original CDS into several protein-coding regions.

An option in the GeneMarkS-T command line allows output to display just one predicted CDS—the one with the largest log-odds score. In terms of predicting the true gene this approach works well for reference and ‘concordant’ transcripts. However, in practical cases of new assembled transcripts, how should one know to which class a given transcript belongs? Another command line option generates all the predicted CDS with their scores. In this case, post-processing would select transcripts with single and, as we have demonstrated, reliable predictions. Transcripts with multiple predictions require further analysis to classify them into those with and without assembly errors (about 90 and 10% respectively, based on our observations).

Assessment of gene prediction accuracy in assembled transcripts must be limited to transcripts with protein coding regions not disrupted by assembly errors, i.e. the ‘concordant’ transcripts (see ‘Materials and Methods’ section). Tests of GeneMarkS-T, Prodigal and TransDecoder on five sets of *D. melanogaster* ‘concordant’ transcripts showed that GeneMarkS-T delivered more accurate predictions (Table 3). This observation was in agreement with the results of accuracy assessment on reference transcripts (Figures 2 and 3).

We should note that the GeneMarkS-T *mgl* threshold of 150 bp was the lowest among the three gene finders (Table 3). Prodigal (TransDecoder) required the filtering of predictions shorter that 210 bp (270 bp). The ability to choose a lower threshold indicates that GeneMarkS-T works more accurately in the short gene range than other tools.

We used the data generated by the novel Ribo-seq technique (12) to prepare a test set of mouse transcripts with validated gene starts. While the number of correctly predicted CDS, counted by the correct gene stops was quite close to the numbers detected by TransDecoder and Prodigal, GeneMarkS-T generated a significantly fewer number of false positives; it also demonstrated better accuracy in TIS prediction (Table 2).

We observed that GeneMarkS-T was able to work even with a very small volume of sequence (Figure 4), down to a single transcript, where it automatically switched to use of heuristic models (13^15^). This ability makes GeneMarkS-T suitable to analyse transcripts in metatranscriptomes, an application not immediately appropriate for other gene finders. The results (the last row in Table 3) show that this option, initiated from the command line, delivers surprisingly good accuracy for transcripts ranging in G + C content from 27 to 63%.

## SOFTWARE AVAILABILITY

The GeneMarkS-T software is freely available for academic research and can be downloaded from http://topaz.gatech.edu/GeneMark/license_download.cgi.

## SUPPLEMENTARY DATA

Supplementary Data are available at NAR Online.

SUPPLEMENTARY DATA
