# Supplementary material for: Identification of protein coding regions in RNA transcripts
Source: Nucleic Acids Res. 2015 Apr 13;43(12):e78. doi: 10.1093/nar/gkv227 (PMC4499116; doi:10.1093/nar/gkv227)
Supplement: SUPPLEMENTARY DATA [file supp_43_12_e78__index.html]

Identification of protein coding regions in RNA transcripts — SUPPLEMENTARY DATA 

# Identification of protein coding regions in RNA transcripts

## SUPPLEMENTARY DATA

**Files in this Data Supplement:**

- SUPPLEMENTARY DATA
