# Supplementary material for: Identification of protein coding regions in RNA transcripts
Source: Nucleic Acids Res. 2015 Apr 13;43(12):e78. doi: 10.1093/nar/gkv227 (PMC4499116; doi:10.1093/nar/gkv227)
Supplement: SUPPLEMENTARY DATA [file supp_gkv227_nar-01659-met-g-2014-File008.doc]

**Supplementary Materials**

**Identification of Protein Coding Regions in RNA Transcripts**

Shiyuyun Tang^1^, Alexandre Lomsadze^2^, and Mark Borodovsky^2,3,4^*

^1^ School of Biology, ^2^ Joint Georgia Tech and Emory Wallace H. Coulter Department
 of Biomedical Engineering, ^3^ School of Computational Science and Engineering,
^4^ Center for Bioinformatics and Computational Genomics,
Georgia Institute of Technology, Atlanta, GA, 30332, USA


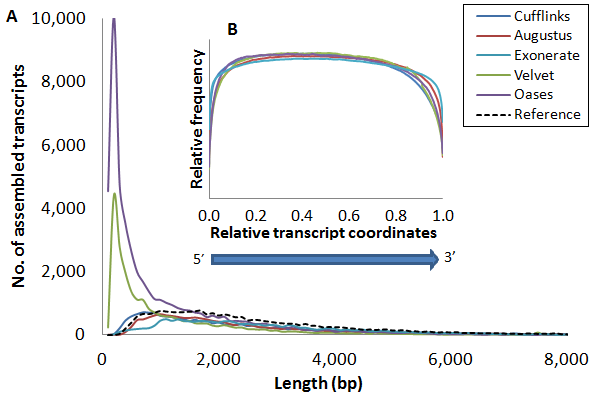


**Figure S1.** (A) Length distributions of reference *D.* *melanogaster* transcripts (RefSeq transcripts) as well as transcripts reconstructed from RNA-Seq reads by Cufflinks, Augustus, Exonerate, Velvet and Oases; (B) Frequency of observing particular transcript section being present in assembled transcripts (shown in relative transcript coordinates). Here top values of the relative frequency are close to 1.0. Note that this graph should not have an integral under the curve equal to one as the transcript sections are arguments for separate random variables (present, not-present).


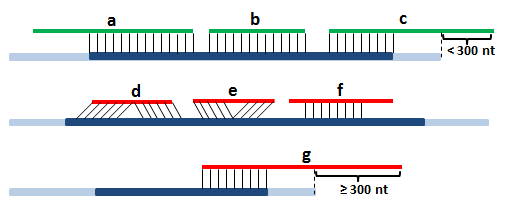


**Figure S2**. Examples of concordant (green) and conflicting (red) transcript assemblies. ‘Concordant’ transcripts have ungapped BLASTn alignments to reference CDS (dark blue) (a-c); extension beyond the limit of reference UTR is not longer than 300bp (c). ‘Conflicting’ transcripts are those that contain gaps in alignment to reference CDS (d-f) or/and have a long extension (> 300bp) beyond reference UTR (g).


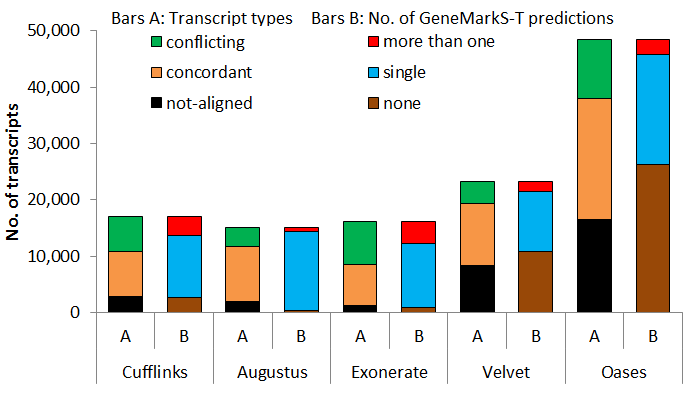


**Figure S3.** Numbers of the three types of assembled transcripts (concordant, conflicting, and not-aligned) as observed in sets of *D. melanogaster* transcripts assembled by the five methods (depicted in bars A). Numbers of the three types of events: GeneMarkS-T predicting i/ more than one, ii/ single and iii/ none coding regions, in *D. melanogaster* reference transcripts (depicted in bars B). Predicted coding regions with length less than 300bp were discarded.


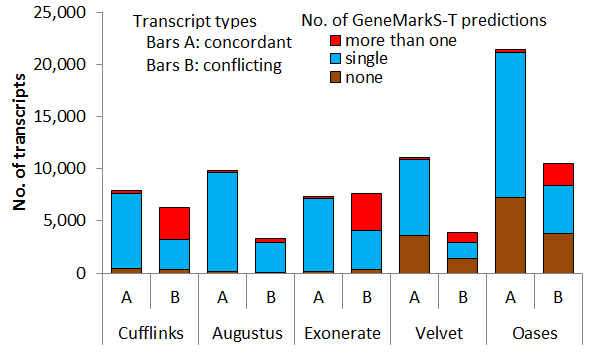


**Figure S4.** Numbers of the three types of events: GeneMarkS-T predicting i/ more than one, ii/ single and iii/ none coding regions, in *D. melanogaster* concordant (bars A) and conflicting transcripts (bars B). The *mgl* value was 300bp. Events of prediction of multiple coding regions were registered prior to selecting ‘reported’ predictions with highest log-odd score.

**Table S1. Characteristics of GeneMarkS-T accuracy of gene predictions in reference transcripts of *M. musculus* and *D. melanogaster*. GeneMarkS-T was self-trained with or without dividing transcripts into more G+C homogeneous sets (clusters). The borders of the three clusters were set as .31, .46, .52 and .76 for *M. musculus* and .27, .48, .51 and .63 for *D. melanogaster.* The *mgl* value was 300bp.**

| Species | # of clusters | TP | FP | Sn | Sp |
| --- | --- | --- | --- | --- | --- |
| *D. melanogaster* | 1 | 12,007 | 370 | 90.7 | 97.0 |
|  | 3 | 12,236 | 374 | 92.4 | 97.0 |
| *M. musculus* | 1 | 18,346 | 303 | 96.9 | 98.4 |
|  | 3 | 18,380 | 269 | 97.1 | 98.6 |

**Table S2. Numbers of the three types of events: predicting i/ more than one, ii/ single and iii/ none coding regions by GeneMarkS-T, Prodigal, and TransDecoder in *D. melanogaster* transcripts of concordant type reconstructed from RNA-Seq reads by Cufflinks, Augustus, Exonerate, Velvet and Oases. The *mgl* value was 300bp.**

| Assembly method | # of concordant transcripts | Prediction tool | # of predicted coding regions | | |
| --- | --- | --- | --- | --- | --- |
|  |  |  | >1 | 1 | 0 |
| Cufflinks | 7,886 | GeneMarkS-T | 236 | 7,220 | 430 |
|  |  | Prodigal | 184 | 7,188 | 514 |
|  |  | TransDecoder | 483 | 6,828 | 575 |
| Augustus | 9,834 | GeneMarkS-T | 191 | 9,446 | 197 |
|  |  | Prodigal | 139 | 9,431 | 264 |
|  |  | TransDecoder | 502 | 9,017 | 315 |
| Exonerate | 7,375 | GeneMarkS-T | 231 | 6,971 | 173 |
|  |  | Prodigal | 189 | 6,985 | 201 |
|  |  | TransDecoder | 537 | 6,612 | 226 |
| Velvet | 11,032 | GeneMarkS-T | 135 | 7,320 | 3,577 |
|  |  | Prodigal | 109 | 7,244 | 3,679 |
|  |  | TransDecoder | 324 | 6,967 | 3,741 |
| Oases | 21,409 | GeneMarkS-T | 306 | 13,830 | 7,273 |
|  |  | Prodigal | 297 | 13,653 | 7,459 |
|  |  | TransDecoder | 696 | 13,221 | 7,492 |
